# Policy framework for the utilization of generative AI

**DOI:** 10.1186/s13054-024-04917-z

**Published:** 2024-04-18

**Authors:** Kunming Cheng, Haiyang Wu

**Affiliations:** 1 Department of Intensive Care Unit, The Second Affiliated Hospital of Zhengzhou University, Zhengzhou, China; 2 Department of Orthopaedics, The First Affiliated Hospital of Zhengzhou University, Zhengzhou, China; 3 Department of Clinical College of Neurology, Neurosurgery and Neurorehabilitation, Tianjin Medical University, Tianjin, China

The ascent of generative artificial intelligence (AI) technologies has engendered a pivotal juncture in policy formulation, necessitating a comprehensive framework to govern their deployment [1–3]. Here, we summarized several the use of guidelines for generative AI from different institutions. In May 2023, the International Committee of Medical Journal Editors (ICMJE) issued updated recommendations stipulating that journals should require authors to disclose the use of AI-assisted technologies during the manuscript writing process, such as large language models, chatbots, or image generation tools. Authors utilizing these technologies are expected to elucidate their usage within the cover letter and manuscript. Moreover, authors are prohibited from attributing chatbots (e.g., ChatGPT), AI, or AI-assisted technologies as authors or co-authors. It is imperative that authors ensure the absence of plagiarism in their manuscripts, encompassing both text and images generated by AI. Reviewers are advised against uploading manuscripts to software platforms or other AI technologies incapable of ensuring confidentiality. Furthermore, reviewers should disclose to journals whether and how AI technologies were utilized in the evaluation of manuscripts or the drafting of reviewer comments. The World Association of Medical Editors (WAME) has issued the following recommendations regarding the use of ChatGPT and chatbots in academic publishing: chatbots could not be listed as authors. Authors should maintain transparency when utilizing chatbots and provide information on how they were used. Authors are responsible for the work done by chatbots in the manuscript (including the accuracy and non-plagiarism of the presented content) and must acknowledge all sources, including materials generated by chatbots. Journal editors need appropriate tools to help detect content generated or modified by AI. In addition, On December 21, 2023, the Chinese Ministry of Science and Technology issued regulations on responsible research conduct, stating that: Directly generating funding application materials using generative AI is prohibited. Content generated using generative AI, especially involving key factual and opinion-based content, must be clearly labeled and the generation process explained to ensure authenticity, accuracy, and respect for intellectual property rights. Content marked as generated by AI by other authors should generally not be cited as original literature. Unverified references generated by generative AI are prohibited from direct use. Generative AI cannot be listed as a co-contributor to achievements.

In addition to the guidance of the above-mentioned agencies, recently, one study published by *BMJ* examined whether the top 100 academic publishers and the 100 most cited journals have established guidelines for the utilization of generative AI tools [4]. Among the top 100 publishers, 24 (24%) have issued directives regarding the use of GAI. Notably, 87% of the surveyed journals provided guidelines for generative AI employment, with 96% of publishers and 98% of journals explicitly prohibiting the attribution of authorship to generative AI systems. Regarding the disclosure of generative AI implementation, 75% of publishers and 40% of journals specified the nature of disclosure, albeit with varying placement, encompassing sections such as research methodology, acknowledgments, cover letters, or other segments. It is evident that the recommendations offered by major publishers and journals regarding generative AI usage lack structured consensus or guidelines, thereby manifesting significant heterogeneity and conflicting guidance. This underscores the pressing need for interdisciplinary guidelines pertaining to the utilization of generative AI.

## Data Availability

The raw data of the current study are available from the corresponding author on reasonable request.
